# Miniaturized Method for Chemical Oxygen Demand Determination Using the PhotoMetrix PRO Application

**DOI:** 10.3390/molecules27154721

**Published:** 2022-07-23

**Authors:** Lisandro von Mühlen, Osmar D. Prestes, Marco F. Ferrão, Carla Sirtori

**Affiliations:** 1 Instituto de Química, Universidade Federal do Rio Grande do Sul, Av. Bento Gonçalves, 9500, Porto Alegre 91501-970, RS, Brazil; von.muhlen@ufrgs.br (L.v.M.); marco.ferrao@ufrgs.br (M.F.F.); 2 Laboratório de Análises de Resíduos de Pesticidas (LARP), Departamento de Química, Universidade Federal de Santa Maria, Av. Roraima, 1000, Santa Maria 97105-900, RS, Brazil; osmar.prestes@ufsm.br

**Keywords:** chemical oxygen demand, PhotoMetrix PRO, miniaturization, AGREE score, waste reduction, environmental analysis, photo-Fenton

## Abstract

The analysis of chemical oxygen demand (COD) plays an important role in measuring water pollution, but it normally has a high ecological price. Advances in image acquisition and processing techniques enable the use of mobile devices for analytical purposes. Here, the PhotoMetrix PRO application was used for image acquisition and multivariate analysis. Statistical analysis showed no significant difference in the results compared to the standard method, with no adverse effect of the volume reduction. The cost of analysis and waste generation were reduced by one third, while the analysis time was reduced by one fifth. The miniaturized method was successfully employed in the analysis of several matrices and for the evaluation of advanced oxidation processes. The AGREE score was improved by 25% due to miniaturization. For these reasons, the miniaturized PhotoMetrix PRO method is a suitable option for COD analysis, being less hazardous to the environment due to reductions in the chemicals used and in waste generation.

## 1. Introduction

The global population is expected to increase by 10% in the next decade, reaching 8.5 billion in 2030 [1]. As a consequence, greenhouse gas emissions and waste generation will increase, contributing to environmental hazards [2,3]. Incorrect waste disposal increases the concentrations of organic matter in water bodies. The organic matter content in water is measured by determining the amount of oxygen required during a digestion step. The oxygen demand (the amount of O_2_ required to degrade organic matter) can be divided into biochemical oxygen demand (BOD) and chemical oxygen demand (COD) [4,5,6].

COD analysis, normally using the dichromate oxidation method, plays an important role in the measurement of water pollution, as it is much faster than BOD analysis, but it has a higher ecological price [7]. In addition, the standard method for colorimetric COD analysis is employed in the characterization of wastewaters for the purposes of risk assessment and the development of new wastewater treatment methods, such as advanced oxidation processes [8,9,10,11].

The standard technique uses large amounts of sample and hazardous reagents such as sulfuric acid (H_2_SO_4_), potassium dichromate (K_2_CrO_7_), and mercuric sulfate (HgSO_4_) [12,13,14]. Different modifications of the COD method have been proposed in order to improve its sustainability and to align it with the principles of green chemistry, but all of them use high volumes of samples and reagents and have different matrix interferences [7,15].

Kolb et al. [16] proposed a method free from mercury and dichromate, using KMnO_4_ as anoxidizer, although it required 50 mL of Mn(III) reagent for each test tube, 5 mL ofsample, and a previous chloride precipitation step. Another study avoiding the use of dichromate was presented by Carbajal-Palacios et al. [17], where the oxidizer employed was hydrogen peroxide. The results showed its suitability for COD analysis, but it generated a final waste volume of 75 mL for each sample analyzed. Elsewhere, an amperometric method using nano-Cu/glassy carbon electrode (GCE) sensors was proposed by Badr et al. [18]. While the direct measurement of COD was a remarkable achievement, it was necessary to prepare the nano-Cu/GCE sensors by electrodeposition, as they are not commercially available. An ultrasound-assisted digestion method for COD analysis was developed by Kim et al. [19]. Although the oxidation temperature did not exceed 30 °C and a short total digestion time of only 15 min was required, differences of 5–20% were reported, compared to the standard procedure, and the sonication was not sufficient to digest all of the organic matter in the samples.

Recent advances in image acquisition and processing have led to the use of mobile devices for analytical purposes instead of colorimeters and spectrophotometers [20,21,22]. The PhotoMetrix PRO application enables analyses based on the decomposition of images, together with statistical procedures such as principal component analysis (PCA) and partial least squares (PLS). For this, the images obtained using a smartphone camera can be analyzed using the combination of individually selected color channels or their histograms. The region of interest (ROI) to be analyzed may be previously defined by the user (32 × 32, 48 × 48, 64 × 64, or 96 × 96 pixels). Graphs of the principal component scores and loadings are generated following decomposition of the data matrix [23]. This application has been successfully applied in forensic, water, milk, and gel electrophoresis analyses [24,25,26,27,28].

Another attractive advantage of PhotoMetrix PRO is the ability to use small sample volumes (hundreds of µL), allowing the possibility of miniaturization of the method and improved compliance with the principles of green analytical chemistry. The free AGREE software (Analytical GREEnness metric approach and software, v. 0.4 2020, created by Wojciech Wojnowski (Gdańsk, Poland)) allows users to measure the greenness of a method, on a scale from 0 to 1. It incorporates the 12 SIGNIFICANCE principles of green analytical chemistry as greenness criteria, allows the assignment of weights for each individual criterion, provides a colored pictogram for easy interpretation of weak and strong points, and employs user-friendly GUI software [29].

This study aims at the miniaturization of COD analysis, resulting in smaller amounts of samples and reagents, a shorter analysis time, lower waste generation, and reduced overall costs. In the proposed method, the COD contents of different water matrices were measured using 0.5 mL of dichromate solution, 1.167 mL of silver catalyst solution, and 0.833 mL of sample, leading to a final waste volume of 2.5 mL. The PhotoMetrix PRO application was employed for PLS multivariate analysis, using an ELISA plate in a controlled light environment, enabling the reduction of the analysis time. AGREE software was used to assess the greenness of the miniaturized method compared to the standard colorimetric method. The miniaturized method and standard colorimetric analysis were then compared for the determination of the COD values for samples of hospital wastewater, simulated wastewater, surface water, and underground water, as well as for photo-Fenton process samples (initially and after the treatment), using the PhotoMetrix PRO application.

## 2. Results

### 2.1. Standard Colorimetric Method versus PhotoMetrix PRO Analysis

In order to determine whether the apparatus (described in Section 4.3) was suitable for COD analyses, spiked ultrapure water (UPW) was digested, and the results obtained employing the PhotoMetrix PRO application were compared with those obtained using a spectrophotometer. For the analysis, two aliquots of spiked UPW were prepared at a final concentration of 150 mg L^−1^ O_2_. Digestion and analyses were performed according to APHA Standard Method 5220 D (using 2.5 mL of sample, 1.5 mL of K_2_Cr_2_O_7_ digestion solution, and 3.5 mL of 2.75% (*m*/*v*) Ag_2_SO_4_ solution in H_2_SO_4_) [14]. The standard colorimetric method provided an average value of 149.4 ± 3mg L^−1^ O_2_, while the PhotoMetrix PRO method provided an average value of 153.0 ± 20 mg L^−1^ O_2_. These results showed that the PhotoMetrix PRO results were comparable with those obtained with the standard colorimetric method, in accordance with a previous study [30].

Since the two methods presented similar results for the spiked samples, tests were performed with the volumes reduced by one third. The miniaturized method employed 0.833 mL of sample, 0.500 mL of K_2_Cr_2_O_7_ digestion solution, and 1.167 mL of 2.75% (*m*/*v*) Ag_2_SO_4_ solution in H_2_SO_4_. For the analysis, ultrapure water was spiked to a final concentration of 150 mg L^−1^ O_2_. The analyses were performed on three different days (total *n* = 6), and statistical F and *t*-tests were performed at the 95% confidence level. The analytical results(*n* = 6) were 145 ± 11 mg L^−1^ O_2_(RSD = 7.6%)for the analyses employing the PhotoMetrix PRO application and 138 ± 10 mg L^−1^ O_2_(RSD = 7.2%) for the standard colorimetric method. Table 1 presents the validation parameters for each method. The limits of detection (LOD) and quantification (LOQ) were calculated according to the INMETRO guidelines [31]. It should be noted that the concentration representing the first point of the calibration curve (60 mg L^−1^) was adopted as the experimental LOQ for both validation methods.

The miniaturized method was applied for the analysis of different water samples. Prior to the analysis, the hospital wastewater (HWW), simulated wastewater (SWW), groundwater (GW), and surface water (SW) samples were filtered using qualitative filter paper. The HWW and SWW samples were diluted with ultrapure water in a proportion of 1:2 (*v*/*v*). The SW sample was diluted with ultrapure water in a proportion of 1:1 (*v*/*v*). A tap water (TW) sample was spiked to a final theoretical O_2_ concentration of 150 mg L^−1^ O_2_ to ensure that the results would be within the calibration range. UPW was also spiked with 150 mg L^−1^ O_2_. The results are shown in Table 2.

Advanced oxidation processes (AOPs) aim at the degradation of biorefractory or hazardous organic compounds in wastewater. During the process, organic compounds are oxidized by free radicals, especially the hydroxyl radical (•OH), and in some cases are mineralized to water, carbon dioxide, and inorganic ions. The photo-Fenton process produces hydroxyl radicals using the combination of Fe(II) or Fe(III) ions, H_2_O_2_, and UV radiation [32]. As a result of the mineralization, the COD content of wastewater is reduced during the photo-Fenton process, so this is a useful parameter for following the degradation promoted by the treatment and for evaluating the process efficiency [33]. Furthermore, COD is also an essential parameter used for determination of the average oxidation state (AOS) of wastewater during treatment. The AOS parameter relates the dissolved organic carbon (DOC) and COD (expressed in C L^−1^ and O_2_ L^−1^, on a molar basis, respectively) for samples obtained during the treatment process [34]. The AOS may be between +4 for CO_2_ (more oxidized state of C) and −4 for CH_4_ (less oxidized state of C). As indicated in previous work, the AOS usually increases with treatment time, until reaching a plateau. In this step, chemical treatment only mineralizes organic contaminants, without partial oxidation [35].

**Figure 1:**
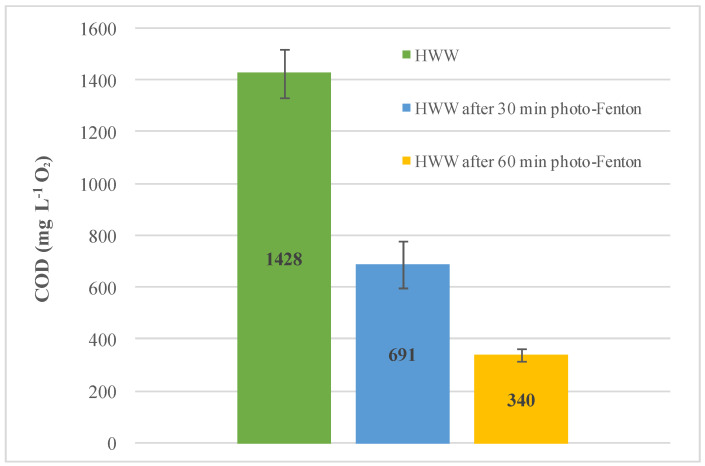
Results for COD analysis of spiked HWW during photo-Fenton treatment, using the miniaturized PhotoMetrix PRO method, at t = 0, t = 30, and t = 60 min.

In the present study, the miniaturized colorimetric method was employed in COD monitoring of HWW during a photo-Fenton process. The COD analysis was performed for the raw HWW sample before starting the photo-Fenton treatment, after 30 min of the process, and at the end of 60 min of treatment (the results are shown in Figure 1). As expected, it was possible to observe the reduction in the COD content during the photo-Fenton process employing the proposed method.

### 2.2. Cost and Time Reductions and Greenness Improvement Due to Analysis Miniaturization

For the purpose of this study, the analysis cost was estimated considering only the reagent costs. The hardware devices were not taken into account, as they have a long service life if used with care. Other operating costs, such as analyst working hours, were also not considered. As shown in Table 3, the total analysis cost decreased by one third.

AGREE software enables users to calculate the compliance of a method with the 12 SIGNIFICANCE principles of green analytical chemistry. It converts the 12 principles into scores, providing a metric tool for the assessment of greenness of analytical procedures, on a scale from 0 to 1. The software converts the scores for each individual criterion, and the final assessment result is the product of the assessment results for each principle. The output is a clock-like graphic, with the final score in the middle and 12 segments surrounding it, representing the 12 SIGNIFICANCE principles. The performance for each criterion is indicated using a red–yellow–green scale (the greener the better), and the weight of each principle is indicated by the width of each segment [29].

**Figure 2:**
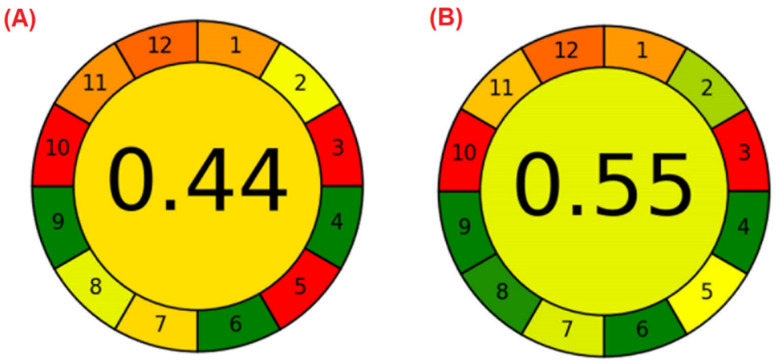
AGREE score results for (**A**) the standard colorimetric method and (**B**) the miniaturized method.

When comparing two methods, the overall score in the middle closer to 1 and in greener color indicates the greener procedure. Figure 2 shows the calculated results for the standard colorimetric method (A) and the miniaturized method (B). The AGREE software allow users to assign weights to each criterion. As a way to ensure unbiased results, the weights of all 12 principles were set as 1 for the AGREE score calculation. Table 4 provides details of the 12 criteria measured by the AGREE software and the set of parameters used for the greenness score calculation for each evaluated method. The values of the parameters for criteria 1, 3, 4, 5, 9, 10, and 12 were selected from the data sets provided in the software.

## 3. Discussion

The spiked UPW samples showed a deviation lower than 10% from the expected value. In the application of the statistical F-test, the value of F was lower than F_critical_, indicating that there was no significant difference between the results for the two techniques, showing that the miniaturization of the method did not affect its precision. In the statistical *t*-test, the value of t was lower than t_critical_, demonstrating that the decrease in volume did not affect the results. There was no significant difference in accuracy, as the comparative analysis of COD over a period of three days (*n* = 6) resulted in RSD = 7.6% and RSD = 7.2% for the miniaturized PhotoMetrix PRO method and the standard colorimetric method, respectively.

Regarding the time required, both methods could be divided into two steps: the digestion step and the analytical step. The time required for the digestion step was exactly the same for both methods, as in both cases it was necessary to pipette the volumes of the reagents and sample, followed by the digestion itself.

On the other hand, the time required for the analytical step was significantly reduced using the PhotoMetrix PRO method, as after transferring the aliquots to the ELISA plate, it was only necessary to align them to the focus of the camera. In the case of the colorimetric method, it was necessary to clean and refill the cuvette between each analysis. In terms of the analysis time, the PhotoMetrix PRO method was five times faster than the standard colorimetric method. While 30 min were required to analyze sic samples using the standard colorimetric method, the same number of samples could be analyzed in 6 min using the miniaturized method.

Table 5 presents a comparison of advantages and disadvantages for the miniaturized PhotoMetrix PRO method and some other modified methods for COD analysis. While the proposed method still uses dichromate and mercury, the total amount of reagents needed, sample volume, and waste generated are more than 20 times lower compared to the permanganate and peroxide methods. It is not possible to perform direct analysis, such as can be achieved using the nano-Cu/GCE sensors, but all of the reagents and equipment used in the miniaturized method are commercially available, ensuring its reproducibility and reducing the time needed to prepare the equipment. In the case of the miniaturized method, once the apparatus is set up, it can be used for innumerable analyses, while the copper-based sensors presented a 9% response decrease after seven days. In comparison with ultrasound-assisted digestion, the miniaturized method requires a longer digestion time and higher temperature, but the digestion is sufficient to digest all of the organic matter present in the sample, as it uses the same ratio between reagents and samples as the standard method.

According to the results obtained with AGREE software, the use of the miniaturized method provided a 25% improvement in the greenness of the COD analysis. Although the overall factor was still not optimal (score 1 on the software scale), it nonetheless represented an important improvement to the method, considering the nature of the reagents used and the widespread use of COD analysis for monitoring wastewater samples. Additionally, parameters 2, 5, 7, and 8 were those that showed the greatest changes and most reflected the greener potential of the miniaturized COD method. Therefore, the miniaturized methodology evaluated here provided partial compliance with the principles of green analytical chemistry, with miniaturization enabling reductions in the amounts of reagents employed in the measurement step, resulting in decreases of some environmental, human, and financial impacts [36,37].

## 4. Materials and Methods

### 4.1. Chemicals and Water Matrices

The COD analyses were performed according to Standard Method 5220 D [14]. All reagents were analytical grade, with a purity of at least 97.2%. H_2_SO_4_ was purchased from Neon (Brazil). HgSO_4_ was purchased from Quimibrás (Brazil). Silver sulfate (Ag_2_SO_4_) was obtained from Nuclear (Brazil). K_2_Cr_2_O_7_ and magnesium sulfate (MgSO_4_·7H_2_O) were purchased from Synth (Brazil). Potassium hydrogen phthalate (KHP), calcium chloride (CaCl_2_·2H_2_O), and potassium phosphate (K_2_HPO_4_) were purchased from Dinâmica (Brazil). Urea (CH_4_N_2_O), bacteriological peptone, and beef extract were purchased from KASVI (Brazil).

Prior to use, the K_2_Cr_2_O_7_and KHP were dried for 2 h at 150 °C and 110 °C, respectively. The KHP stock solution (425.0 mg L^−1^) was prepared in ultrapure water. Working standard solutions were prepared in class A glassware, by appropriate dilution of the stock solution. A stock digestion solution of K_2_Cr_2_O_7_ was prepared at a concentration of 10.216 g L^−1^, containing 33.300 g L^−1^ HgSO_4_, in 16% (*v*/*v*) H_2_SO_4_ solution. The stock solutions and the working standards were stored in amber flasks under refrigeration.

The experiments were performed using six water matrices: UPW, HWW, SWW, GW, SW, and TW. The HWW was collected from a public hospital located in the city of Porto Alegre (Rio Grande do Sul, Brazil). HWW is well known as a complex matrix, generally characterized by a large content of pathogens, pharmaceutical compounds, and their metabolites, biological fluids, and excrement [38]. The use of SWW enables reproducible simulation of the organic and ionic contents of hospital wastewaters, as a matrix free from pathogens, pharmaceuticals, and metabolites [39]. The SWW was prepared using peptone, beef extract, urea, MgSO_4_·7H_2_O, and CaCl_2_·2H_2_O, as described by Lumbaque et al. [40]. SW and GW were collected from agricultural sites in the municipalities of Teutônia (Rio Grande do Sul, Brazil) and Caxias do Sul (Rio Grande do Sul, Brazil). Organic matter is naturally present in surface water as a result of the breakdown of terrestrial plants and byproducts of bacteria, algae, and aquatic plants [41]. Another source of organic matter in surface water is the incorrect disposal of wastes from urban, industrial, and agricultural activities [42]. The incorrect disposal of liquid and solid waste can also lead to the infiltration of water-soluble compounds into underground water sources, while pumping may cause the migration of contaminated water from the surrounding area to wells [43]. The HWW, SWW, GW, and SW samples were filtered using qualitative filter paper to remove any solid content. Ultrapure water (18.2 MΩ·cm) was obtained from a Milli-Q^®^ system (Millipore, Billerica, MA, USA).

### 4.2. Sample Digestion and Standard Colorimetric Method

The standard colorimetric method for COD analysis had a final volume of 7.5 mL (2.5 mL of sample, 1.5 mL of K_2_Cr_2_O_7_ digestion solution, and 3.5 mL of 2.75% (*m*/*v*) Ag_2_SO_4_ solution in H_2_SO_4_). The COD content of the sample was calculated using a calibration curve constructed with different concentrations of KHP to simulate O_2_ concentrations, where 1 mg of KHP corresponds to 1.171 mg of O_2_. The KHP stock solution had a theoretical O_2_ content of 497.6 mg L^−1^. The samples and standards were digested for 2 h in closed 10 mL vessels at 150 °C. Since the selected environmental aqueous matrices presented high COD values, the miniaturized method was only tested for the high range of calibration curve concentrations, from 60 to 450 mg L^−1^ [14].

For standard colorimetric analysis, a Cary 50 UV–Vis spectrophotometer (Agilent Technologies, Santa Clara, CA, USA) was used, with a 1 cm path length quartz cuvette, at λ = 600 nm. The calibration curve was prepared using 10.216 g L^−1^ K_2_Cr_2_O_7_ digestion solution, with 6 calibration points obtained by dilution of the KHP stock solution. The KHP concentration range was from 60 to 450 mg L^−1^ O_2_, covering typical COD levels in wastewaters, with the possibility of dilution if levels exceeded the upper limit.

### 4.3. PhotoMetrix PRO Analysis

**Figure 3:**
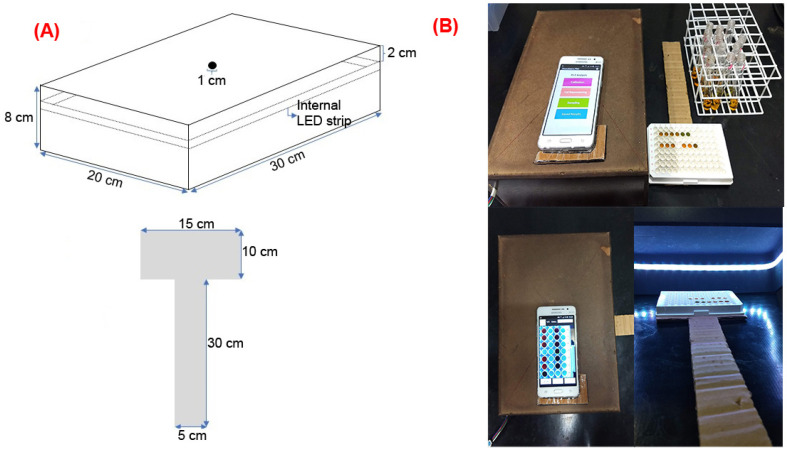
Design of the apparatus used for the miniaturized analysis (**A**) and the apparatus in use during COD analysis (**B**).

PhotoMetrix PRO was used for PLS regression, employing a Samsung Galaxy J5 smartphone (Samsung, Brazil) operating with Android 5.1.1 and equipped with a 13MP camera, with ROI of 32 × 32 pixels. The device was supported on an MDF box with a central hole for camera lens access, a black interior, and an RGB 5050 LED strip as a white light source within the box (Figure 3A). Additional information about the PhotoMetrix PRO system may be found at https://youtu.be/Su9x4uqNEHo (accessed on 10 February 2021).

A 144-well ELISA microplate was used to support 340 µL aliquots of digested samples and standards. The design of the MDF box with the inner LED strip ensured homogeneous illumination of the ELISA plate, reducing the error caused by indirect light. A cardboard support (Figure 3A) was used to move the plate inside the MDF box in order to position the wells in the field of view of the camera. The apparatus used for the miniaturized COD analysis is shown in Figure 3B.

The analysis using PhotoMetrix PRO was performed with the same apparatus described previously in order to enable comparison of the two procedures. The spectrophotometric analysis was performed using the same equipment and type of cuvette.

### 4.4. COD Method Miniaturization

The final volume was 2.5 mL (1/3 of the original volume). The volumes of all the reagents, samples, and standard solutions were reduced by the same proportion(0.5 mL of K_2_Cr_2_O_7_ solution, 1.167 mL of Ag_2_SO_4_ solution, and 0.833 mL of sample or standard solution).

The analysis using PhotoMetrix PRO was performed with the apparatus described previously. To enable comparison between the two procedures, the spectrophotometric analysis was performed using the same equipment and cuvette mentioned above.

## 5. Conclusions

The miniaturized method using PhotoMetrix PRO is a viable and attractive option for performing COD analyses. The results were not adversely affected by the volume reduction, and lower LOD and LOQ values were achieved using the miniaturized method. There was no change in the expected interferences, as the ratio between the reagents and the sample was the same as that in the standard method.

The miniaturized method was successfully employed in the analysis of urban wastewater (HWW), synthetic wastewater (SWW), and environmental matrices (SW and GW), as well as for monitoring the photo-Fenton treatment of HWW. Reductions were obtained in terms of cost and time, as well as in the generation of toxic waste and vapor. The overall cost for COD determination was reduced by one third, while it was possible to reduce the analysis time by one fifth of that required for the standard colorimetric method. In comparison with other modified methods for COD analysis, the proposed method generates up to 20 times less waste, the apparatus is much more accessible, and the use of the same reagents as in the standard colorimetric method, in the same proportions, ensures the total oxidation of the organic matter present in the sample. The volume reduction implies a three-fold lower generation of waste containing heavy metals (Cr, Hg, and Ag) and sulfuric acid, resulting in a much more environmentally friendly method.

In addition, the generation of vapors during the digestion step was reduced, as less sulfuric acid was needed, so the working environment became less hazardous, in accordance with the principles of green analytical chemistry. All of these improvements led to a greener method, as indicated by the 25% improvement in the AGREE score.

## Figures and Tables

**Table 1:** Regression and validation parameters for the standard colorimetric method and the PhotoMetrix PRO method.

Parameter	Standard Colorimetric Method	Miniaturized Method (PhotoMetrix PRO)
Range	60–450 mg L^−1^	
Slope	0.0002	1.4035
Intercept	0.052	0.001
Regression coefficient (r²)	0.9932	0.9999
LOD (mg L^−1^)	9.42	2.28
*LOQ (mg L^−1^)	60	

* Defined as the lowest concentration point used in the calibration curve.

**Table 2:** Results of analyses using the standard colorimetric method and the miniaturized PhotoMetrix PRO method for determination of COD in various environmental aqueous matrices, showing the deviation between the methods.

Sample	COD Results (mg L^−1^ O_2_)
HWW	442 ± 11
SWW	399 ± 60
GW	243 ± 6
SW	616 ± 28
Spiked TW	166 ± 9
Spiked UPW	150 ± 8

**Table 3:** Costs of consumables used for COD analysis: cost per item per sample, total cost per sample, and total cost for the analysis of 10 samples.

		Cost per Sample Analysis ($US)	
Item	Value ^(a)^	Standard Colorimetric Method	Miniaturized Method
K_2_Cr_2_O_7_	982.00/kg	0.0150	0.0050
Ag_2_SO_4_	7360.00/kg	0.2570	0.0859
H_2_SO_4_	493.42/L	1.85	0.6168
HgSO_4_	694.98/kg	0.0347	0.0116
KHP	330.69/kg	0.0008 ^(b)^	0.0003 ^(b)^
**Total cost for 1 sample ($US) ^(c)^**		15.11	5.05
**Total cost for 10 samples ($US) ^(c)^**		34.53	11.51

^(a)^ All values considered were for analytical-grade reagents. ^(b)^ Cost considering the preparation of a 6-point calibration curve. ^(c)^ Cost considering digestion to obtain a 6-point calibration curve.

**Table 4:** The twelve criteria measured by the AGREE software and the set of parameters used for the greenness score calculation for each method evaluated.

Criteria	Standard Colorimetric Method	Miniaturized Method
**1.** Direct analytical techniques should be applied to avoid sample treatment.	Off-line analysis	
**2.** Minimal sample size and minimal number of samples are goals.	2.500 mL sample size	0.833 mL sample size
**3.** In situ measurements should be performed.	Analytical device is positioned off-line	
**4.** Integration of analytical processes and operations saves energy and reduces the use of reagents.	3 or fewer sample preparation steps	
**5.** Automated and miniaturized methods should be selected.	Manual method—not miniaturized	Manual method—none or miniaturized
**6.** Derivatization should be avoided.	Derivatization not needed	
**7.** Generation of a large volume of analytical waste should be avoided and proper management of analytical waste should be provided.	7.5 mL of waste per sample	2.5 mL of wasteper sample
**8.** Multianalyte or multiparameter methods are preferred to methods using one analyte at a time.	1 parameter 12 samples per hour	1 parameter 60 samples per hour
**9.** The use of energy should be minimized (most energy-intensive technique).	UV–Vis spectrometry	Non-instrumental detection
**10.** Reagents obtained from renewable sources should be preferred.	No reagents from bio-based sources	
**11.** Toxic reagents should be eliminated or replaced.	5.000 mL of toxic reagents used	1.666 mL of toxic reagents used
**12.** The safety of the operator should be increased.	Threats not avoided: toxic to aquatic life; bioacumulative; persistent; corrosive	

**Table 5:** Advantages and disadvantages of some modified methods for COD analysis.

Method Description	Advantages	Disadvantages
Miniaturized PhotoMetrix PRO method	Reduced volumes of reagents, sample, and waste generated; more time- and cost-effective.	Use of dichromate and mercury.
Use of KMnO_4_ as an oxidizer [16]	Free of mercury and dichromate.	55 mL of waste generated.
Use of H_2_O_2_ as an oxidizer [17]	Free of dichromate.	75 mL of waste generated. Use of mercury.
Use of nano-Cu/GCE sensors [18]	Direct analysis.	Sensors not commercially available.
Ultrasound-assisted digestion [19]	Reductions of temperature and digestion time.	Sonication not sufficient to digest all of the organic matter. Use of dichromate and mercury.

## Data Availability

The data presented in this study are available on request from the corresponding author.

## Sample Availability

Samples of the compounds are not available from the authors.
